# A randomised controlled trial of acceptance and commitment therapy plus usual care compared to usual care alone for improving psychological health in people with motor neuron disease (COMMEND): study protocol

**DOI:** 10.1186/s12883-022-02950-5

**Published:** 2022-11-15

**Authors:** Rebecca L. Gould, Benjamin J. Thompson, Charlotte Rawlinson, Pavithra Kumar, David White, Marc A. Serfaty, Christopher D. Graham, Lance M. McCracken, Matt Bursnall, Mike Bradburn, Tracey Young, Robert J. Howard, Ammar Al-Chalabi, Laura H. Goldstein, Vanessa Lawrence, Cindy Cooper, Pamela J. Shaw, Christopher J. McDermott

**Affiliations:** 1 Division of Psychiatry, University College London, Wing B, 6th floor Maple House, 149 Tottenham Court Rd, W1T 7NF London, UK; 2 Clinical Trials Research Unit, School of Health and Related Research, University of Sheffield, Sheffield, UK; 3 Priory Hospital North London, London, UK; 4 School of Psychology, Queen’s University Belfast, Belfast, Northern Ireland; 5 Department of Psychology, Uppsala University, Uppsala, Sweden; 6 School of Health and Related Research, University of Sheffield, Sheffield, UK; 7 Maurice Wohl Clinical Neuroscience Institute, King’s College London, London, UK; 8 Department of Psychology, Institute of Psychiatry, Psychology & Neuroscience, King’s College London, London, UK; 9 Health Services & Population Research Department, Institute of Psychiatry, Psychology & Neuroscience, King’s College London, London, UK; 10 Sheffield Institute for Translational Neuroscience, University of Sheffield, Sheffield, UK

**Keywords:** Motor neuron disease, Acceptance and Commitment Therapy, Psychological health, Quality of life, RCT

## Abstract

**Background:**

Motor neuron disease (MND) is a rapidly progressive, fatal neurodegenerative disease that predominantly affects motor neurons from the motor cortex to the spinal cord and causes progressive wasting and weakening of bulbar, limb, abdominal and thoracic muscles. Prognosis is poor and median survival is 2–3 years following symptom onset. Psychological distress is relatively common in people living with MND. However, formal psychotherapy is not routinely part of standard care within MND Care Centres/clinics in the UK, and clear evidence-based guidance on improving the psychological health of people living with MND is lacking. Previous research suggests that Acceptance and Commitment Therapy (ACT) may be particularly suitable for people living with MND and may help improve their psychological health.

**Aims:**

To assess the clinical and cost-effectiveness of ACT modified for MND plus usual multidisciplinary care (UC) in comparison to UC alone for improving psychological health in people living with MND.

**Methods:**

The COMMEND trial is a multi-centre, assessor-blind, parallel, two-arm RCT with a 10-month internal pilot phase. 188 individuals aged ≥ 18 years with a diagnosis of definite, laboratory-supported probable, clinically probable, or possible familial or sporadic amyotrophic lateral sclerosis, and additionally the progressive muscular atrophy and primary lateral sclerosis variants, will be recruited from approximately 14 UK-based MND Care Centres/clinics and via self-referral. Participants will be randomly allocated to receive up to eight 1:1 sessions of ACT plus UC or UC alone by an online randomisation system. Participants will complete outcome measures at baseline and at 6- and 9-months post-randomisation. The primary outcome will be quality of life at six months. Secondary outcomes will include depression, anxiety, psychological flexibility, health-related quality of life, adverse events, ALS functioning, survival at nine months, satisfaction with therapy, resource use and quality-adjusted life years. Primary analyses will be by intention to treat and data will be analysed using multi-level modelling.

**Discussion:**

This trial will provide definitive evidence on the clinical and cost-effectiveness of ACT plus UC in comparison to UC alone for improving psychological health in people living with MND.

**Trial registration:**

ISRCTN Registry, ISRCTN12655391. Registered 17 July 2017, https://www.isrctn.com/ISRCTN12655391.

Protocol version: 3.1 (10/06/2020).

**Supplementary Information:**

The online version contains supplementary material available at 10.1186/s12883-022-02950-5.

## Introduction

### Background and rationale

Motor neuron disease (MND) is a rapidly progressive, fatal neurodegenerative disease that predominantly affects motor neurons from the motor cortex to the spinal cord and causes progressive wasting and weakening of bulbar, limb, abdominal and thoracic muscles. Prognosis is poor and median survival is 2–3 years following symptom onset: only 4–10% survive more than 10 years [1–3]. There is no cure, and riluzole, the sole disease-modifying UK-licensed drug, prolongs median survival for only 2–3 months at 1 year [4]. Given the nature and impact of MND symptoms on daily life and the poor prognosis, psychological distress is relatively common in people living with MND (plwMND). Prevalence rates of up to 44% for depression and 30% for anxiety have been observed, and MND has been reported to be the most frequent cause of assisted suicide [5–7]. Although shorter survival times, poorer quality of life and increased risks of suicide and mortality have been reported in those experiencing psychological distress [8–12], clear evidence-based guidance on improving the psychological health of plwMND within the UK is lacking.

Formal psychotherapy is not routinely part of standard care within services for plwMND in the UK. While the value of informal psychosocial support is highlighted in NICE MND guidelines, particular psychological therapies or approaches are not specified [13]. PlwMND may be able to access formal psychological therapies such as Cognitive Behavioural Therapy (CBT) through Improving Access to Psychological Therapy services [14]. However, typically these cannot meet their specific psychological, physical and communication needs in a timely fashion due to issues such as the rapid disease course and mobility problems limiting access. Furthermore, therapists within Improving Access to Psychological Therapy services frequently lack knowledge of and familiarity with MND – an important factor that was highlighted in our previous qualitative work that examined plwMND’s preferences for psychological interventions [15].

Based on our previous findings [15], we developed and adapted a manualised Acceptance and Commitment Therapy (ACT) intervention for the specific psychological, physical and communication needs of plwMND. ACT is an acceptance-based behaviour therapy [16] with a strong evidence base for improving outcomes (such as functioning, quality of life and mood) in chronic pain [17], and a growing evidence base in chronic disease and mental health contexts [18]. It is an alternative form of psychological therapy to traditional therapies such as CBT, taking a different approach to difficulties and using different therapeutic techniques [19]. CBT is focused on alleviating distress or symptoms, and involves changing how one thinks and behaves in emotional situations. It is conventionally offered for common mental health problems following NICE clinical guidelines [14]. In contrast, ACT is focused on increasing personally meaningful behaviour in the presence of distress and symptoms (though distress or symptoms may improve as a by-product of therapy). It uses a variety of methods to increase a person’s willingness to experience uncomfortable or difficult thoughts and feelings so that they can engage in personally meaningful behaviour. These methods include helping people to be more: i) open to and accepting of their difficult internal experiences rather than struggling with them; ii) aware of their experiences and focused on the here-and-now rather than engaging in excessive worry or rumination; and iii) committed to engaging in behaviour guided by their personal values rather than the things they want to avoid.

It has been argued that ACT may be particularly suited to improving outcomes in objectively difficult or immutable situations, such as life-limiting illnesses and chronic conditions [20–24]. As there is no cure for MND, helping people to live their life as best they can alongside MND is likely to be a more pragmatic approach than trying to control or get rid of distressing or difficult experiences. Furthermore, there is evidence that ACT processes such as psychological flexibility predict functioning and quality of life in MND [25] and other progressive, incurable and/or life-limiting conditions, including muscle disorders [26–28], Duchenne muscular dystrophy [29] and palliative care populations [30]. Finally, there is emerging preliminary evidence that ACT might have advantages over conventional CBT through improved engagement, retention and durability of effects [31–34].

To our knowledge, there have been no trials of ACT for plwMND to date. We showed that ACT was feasible to deliver and acceptable to plwMND in an open uncontrolled feasibility study [35]. We will now assess the clinical and cost effectiveness of ACT, modified for plwMND, plus usual multidisciplinary care (UC) in comparison to UC alone for improving psychological health in plwMND.

### Objectives

The objectives are to:

1. Establish the clinical and cost effectiveness of ACT plus UC for improving psychological health in plwMND compared to UC alone in an RCT with an internal pilot phase.
2. To evaluate the effect of ACT plus UC for plwMND compared to UC alone on caregivers of plwMND.
3. To examine perceived mechanisms of impact and the context in which the intervention is delivered by collecting qualitative data from plwMND and study therapists.

## Methods

This protocol is reported in accordance with SPIRIT and TIDIER guidelines [36, 37]. Checklists are presented in Supplementary Files 1 and 2, respectively.

### Design

This will be a multi-centre, assessor-blind, parallel, two-arm RCT with a 10-month internal pilot phase to assess the feasibility of referral rates and acceptability of randomisation. The stop/go criteria for progression to the full RCT are defined as recruitment of 71 plwMND (or 0.51 plwMND per site per month), with ≥ 70% of participants in the intervention arm completing at least two sessions.

### Study setting

PlwMND will be recruited from approximately 14 UK MND Care Centres/clinics and via self-referral.

### Eligibility criteria for plwMND

Inclusion criteria:

- 1. Aged 18 years and over.
- 2. A diagnosis of definite, laboratory-supported probable, clinically probable, or possible familial or sporadic ALS (which is diagnostically synonymous with MND [38] using the World Federation of Neurology’s El Escorial criteria [39], and additionally the progressive muscular atrophy and primary lateral sclerosis variants where appropriate investigation has excluded mimics of MND.

Exclusion criteria:

- 1. A current clinical need for any form of gastrostomy feeding or non-invasive ventilation (NIV). A clinical need is defined as the participant being dependent upon percutaneous endoscopic gastronomy (PEG) to meet all their nutrition and hydration needs or meeting NICE criteria for the offer of a trial of NIV, as defined in Sect. 1.14.17 of NICE Clinical Guideline NG42 [13]. Potential participants who use PEG feeds or receive NIV at earlier points in the disease course because of local practice or for reasons other than their MND diagnosis will not be excluded.
- 2. A diagnosis of dementia using standard diagnostic guidelines [40, 41].
- 3. Currently receiving ongoing formal psychological therapy delivered by a formally trained psychologist or psychotherapist, and unwilling to refrain from engaging in such formal psychological therapy during the receipt of ACT.
- 4. Insufficient understanding of English to enable engagement in ACT and completion of screening measures and patient-reported outcome measures.
- 5. Lacking capacity to provide fully informed written consent, verbal consent (for those who cannot provide written consent), or consent via the use of a communication aid.
- 6. A need for treatment for severe psychiatric disorder such as schizophrenia or bipolar disorder, or expressing suicidal ideation with active plans/suicidal behaviours and imminent intent (hereafter defined as reports of plans to end one's life within the next 2 weeks).
- 7. Other medical factors that could compromise full study participation such as intellectual disabilities or severe sensory deficits (e.g. visual blindness).
- 8. Previous participation in Phase 1 of the COMMEND study (an uncontrolled feasibility study).

### Eligibility criteria for caregivers of plwMND

- 1. Aged 18 years and over.
- 2. Primary informal caregiver of a person with MND who has consented to participate in the trial (either living with the person with MND or a close family member or friend). Absence of caregivers to participate in the trial will not exclude plwMND from participating in the trial.

### Eligibility criteria for study therapists

- 1. Aged 18 years and over.
- 2. Study therapists who are involved in delivering the intervention to plwMND in the trial.

## Acceptance and commitment therapy

A detailed breakdown of the ACT intervention is provided in Supplementary File 3. PlwMND will receive up to eight 1:1 sessions of ACT, each lasting up to 1 h, over the course of four months. A minimum of four sessions will be face-to-face (delivered within the MND clinic, GP surgery or participant's home, or via videoconference, depending on patient preference and therapist availability) and up to four will be delivered via online audio material/CDs (followed by therapist support via videoconference, instant messaging, telephone or email, depending on patient preference). In exceptional circumstances, all sessions may be delivered via telephone where videoconference facilities are not available (e.g. due to COVID-19). A phased ending to the sessions will be incorporated such that they will be weekly for the first six sessions and then fortnightly for the last two sessions. Should participants not complete their sessions within four months, they will still be offered the opportunity to complete up to eight sessions and the number of weeks taken to deliver the intervention will be recorded.

The intervention will be delivered by Band 7 or Band 8 clinical psychologists, counselling psychologists, counsellors or psychotherapists with training in Cognitive Behavioural Therapy or accredited Cognitive Behavioural Therapy therapists, with a minimum of 1 year experience in delivering psychotherapy interventions. Therapists will attend a 4-day experientially-based training workshop on the use of ACT in plwMND, supplemented by freely available online ACT resources and copies of the newly-developed participant workbook, therapist manual and online videos. Training will be delivered by members of the research team with expertise and experience in ACT and MND, and will also include two interested members of the Patient/Caregiver Advisory Group, where possible. After completing training and achieving satisfactory competence in ACT delivery, therapists will deliver ACT for plwMND under fortnightly group supervision via telephone/video call from a Band 8 equivalent clinical psychologist or psychotherapist trained in ACT, with a minimum of five years’ experience in delivering this therapy. Therapists will also attend a 1-day top-up training course after 12 months to review and consolidate skills in delivering ACT to plwMND.

## Usual multidisciplinary care

UC will comprise standard care as outlined in NICE Clinical Guideline NG42 for MND [13], as this is currently what is available within UK healthcare services for plwMND. This will include medication for managing MND and MND-related symptoms, treatments for MND-related symptoms (e.g. physiotherapy, non-invasive ventilation and gastrostomy), and equipment and adaptations to aid activities of daily living, communication and mobility. Coordinated care will be delivered by multidisciplinary healthcare professionals within MND and palliative care services and will include access to other services (including clinical psychology and neuropsychology, counselling, social care, respiratory ventilation, palliative care gastroenterology, orthotics, mobility/assistive technology/communication equipment services and community neurological care teams). All of the MND Care Centres/clinics involved as recruiting sites are endorsed by the MND Association, and therefore are audited against the standard of care outlined in NICE Clinical Guideline NG42 [13].

As some variations in UC may occur, this will be monitored using a modified form of the Client Service Receipt Inventory (CSRI) [42]. Participants in the intervention arm will be asked to refrain from engaging in concurrent formal psychological therapies such as CBT during the receipt of ACT as this may lead to conflicts in therapeutic approaches and goals. Other than this, participants will not be actively discouraged from seeking treatment outside of the trial for ethical reasons, but all such interventions will be recorded as part of the modified CSRI.

## Treatment fidelity

All therapy sessions will be recorded using encrypted digital voice recorders in order to monitor adherence to the treatment manual. Ten percent of sessions will be randomly selected and assessed for treatment fidelity by an independent ACT therapist using an adapted form of the ACT Treatment Integrity Coding Manual [43]. The random selection of sessions will be stratified according to therapist, phase of the intervention (early, middle or late), and phase of study recruitment (early, middle or late), as previously recommended [44]. Sessions will be assessed on a regular basis throughout the duration of intervention delivery so that therapists can receive ongoing feedback on their intervention delivery. Audio recordings will be reviewed and necessary actions taken if ACT-inconsistent deviations are identified.

## Outcomes

The primary outcome will be psychological health as measured by the total score on the McGill Quality of Life Questionnaire-R [45] at 6 months post-randomisation (primary endpoint). This is a global measure of quality of life that has good psychometric properties [45], and has been shown to be sensitive to change e.g. it was able to distinguish between days rated as bad, average and good in people with cancer [46, 47]. It has also been validated in plwMND [48, 49]. It consists of 15 items: a single item measuring overall quality of life, and subscales measuring quality of life across 4 domains: Existential (4 items), Psychological (4 items), Physical (3 items), and Social (3 items).

Secondary outcome measures will be as follows:

1. Hospital Anxiety and Depression Scale [50]: A 14-item self-report measure of depression and anxiety, which provides separate scores for depression and anxiety, as well as an overall score. For the purpose of analysis and, following validation in plwMND and subsequent published recommendations [51], a subset of data will be analysed which omits one item on the depression scale that assesses psychomotor retardation and one item on the anxiety scale that assesses restlessness as these overlap with physical symptoms of MND;
2. Acceptance and Action Questionnaire-II [52]: A 7-item self-report measure of psychological flexibility (an ACT-specific coping measure);
3. EQ-5D-5L [53]: A 5-item self-report measure of health-related quality of life, used to calculate utility scores for use in economic evaluations. Each of the 5 items is rated on a 5-point scale from no problems to extreme problems. This will be collected from both plwMND and caregivers;
4. Non-physical adverse events and physical self-harm;
5. ALS Functional Rating Scale-Revised [54]: The self-administered version of a 12-item measure of function that has been developed for plwMND will be used as an indicator of disease progression;
6. Existential and Psychological subscales of the McGill Quality of Life Questionnaire-R [45]^,2^: These subscales have been included as secondary outcome measures as quality of life in MND (and hence psychological health) has been found to be more associated with psychological/existential factors than physical function/strength [49];
7. Survival at 9 months;
8. Satisfaction with Therapy and Therapist Scale-Revised [55]: A 12-item self-report measure of satisfaction with therapy and satisfaction with the therapist, rated on a 5-point scale from 1 (strongly disagree) to 5 (strongly agree). Six items relate to satisfaction with therapy and six to satisfaction with the therapist;
9. Client Service Receipt Inventory (CSRI) [42] modified for plwMND. This information will be extracted from participants' self-reports, GP medical records and/or MND care centre records, with participants' consent;
10. Quality-adjusted life years and resource use to inform the cost-effectiveness analysis;
11. Zarit Burden Interview [56]: A well-validated 22-item self-report measure of caregiver burden, which will only be collected from caregivers.

## Measures of bias

The following measures of bias will be included:

1. Credibility/Expectancy Questionnaire [57]: A 6-item self-report measure that assesses the credibility of the rationale for therapy and expectations about treatment, which has been adapted for plwMND. PlwMND will be asked to rate four items on a 9-point scale from 1 to 9 (lower scores are worse) and 2 items are scored on an 11-point scale from 0 to 100%;
2. ACT Treatment Integrity Coding Manual [43]: A coding system that has been developed to assess treatment integrity in ACT interventions, which has been used in previous RCTs of ACT [58]. In this coding system, the frequency and depth of coverage of major components of ACT, together with overall adherence and overall therapist competence, are rated on a five-point scale from 1 (not at all) to 5 (extensively);
3. Treatment preferences: Prior to randomisation, plwMND will be asked to rate how much they would hope to receive ACT plus UC and how much they would hope to receive UC alone. They will be asked to rate this on a 4-point scale from 0 (not at all) to 3 (completely);
4. Assessment of blindness at 6 and 9 months: Although plwMND will be asked not to reveal their allocation to blind outcome assessors, some plwMND may accidentally reveal this and some outcome assessors may be able to guess this. Consequently, outcome assessors will be asked at 6 and 9 months to guess whether they think the participant was allocated to the intervention or control arm and to indicate how certain they are of this using a 5-point scale from 0 (not sure at all) to 4 (very sure);
5. Contamination in the control arm: Use of pharmacological or psychological therapies in the control arm will be monitored using the CSRI. Additional exploratory data analysis will be undertaken to assess the impact of this, if necessary.

## Qualitative component

PlwMND and study therapists will be asked to anonymously complete a qualitative satisfaction questionnaire at 6 months follow-up and the end of intervention delivery, respectively, to further examine the acceptability and feasibility of ACT and UC. The satisfaction questionnaire for plwMND in the intervention arm will examine satisfaction with ACT and its suitability and relevance to plwMND, perceived benefits and limitations of the intervention, difficulties in implementing the intervention in their everyday lives, and any recommendations for revising the intervention. The version for those in the control arm will assess the acceptability and feasibility of the psychological aspects of their management within UC rather than all aspects of their management. Questions will focus on what kind of psychological support participants felt they needed and what they actually received, what was helpful and what was not, and what other psychological support would have been helpful. The satisfaction questionnaire for therapists will explore how ACT was delivered in practice (e.g. treatment fidelity, ease of delivering ACT for plwMND, difficulty of skills for participants to learn, etc.). If plwMND are unable to complete the written questionnaire (either via post, email or online) due to MND-related difficulties, they will be invited to complete the questionnaire verbally via telephone, videoconference, or face-to-face interview with an independent member of the research team.

### Participant timeline

**Fig. 1:**
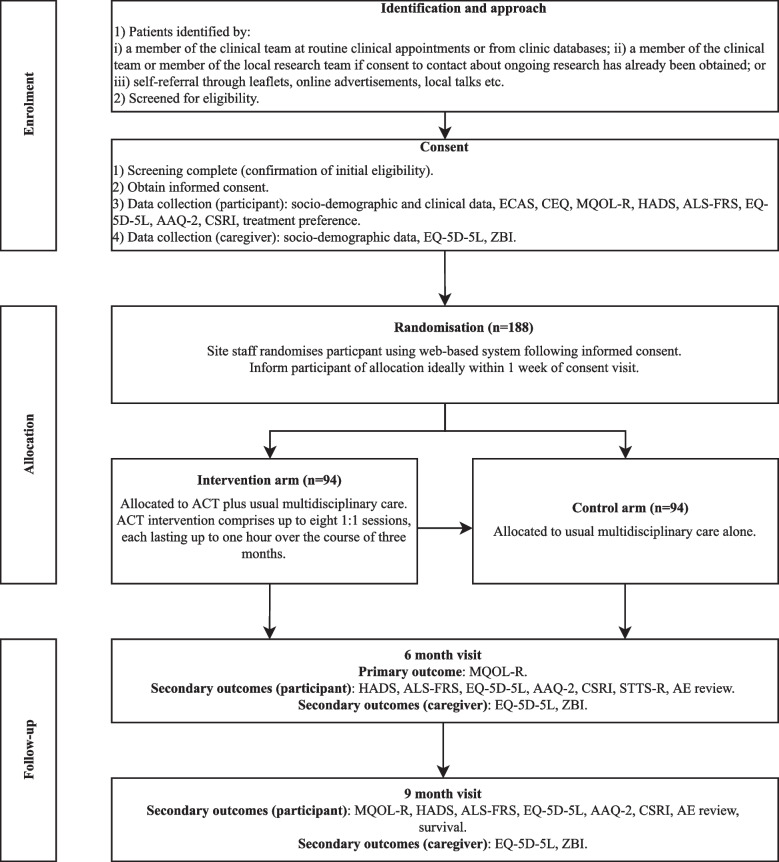
Timeline for plwMND in the trial

Participants will be involved in the RCT for approximately 9 months (± 4 weeks) after randomisation (see Fig. 1).

### Sample size

One hundred eighty-eight plwMND will be recruited from approximately 14 sites. This will allow detection of an effect size of 0.44 standard deviations, with a two-sided alpha of 5% and 90% power. This assumes 20% attrition at 6 months post-randomisation [59], an intra-class correlation coefficient of 0.01 among therapists (as used in other psychotherapy trials [60]) in the intervention arm, assuming 1 therapist per site and a correlation of 0.58 between 0 and 6 months post-randomisation for the McGill Quality of Life questionnaire in plwMND [59]. Our sample size is based on a clinically-meaningful pooled effect size of 0.44 standard deviations reported in a meta-analysis of ACT for mental and physical health conditions vs. controls [61], which falls within the range found for quality of life in studies of ACT in long-term conditions [62]. There are no published data with respect to what a clinically important difference is on the McGill Quality of Life Questionnaire-R in plwMND. However, our effect size is consistent with the minimal clinically important difference of 0.5 standard deviations that has been consistently reported for quality of life across different clinical populations [63].

## Recruitment

Potential participants with MND and their caregivers will be identified and approached about the trial via local clinicians and clinical and research databases and via self-referral through community and online advertisements and talks to local MND Association support groups. Absence of a participating caregiver will not preclude a person with MND taking part in the trial. Once potential participants have been identified, consent for contact will be sought by the clinician (either verbally or with the use of a communication aid), where necessary. If consent is obtained, a member of the local research team will contact the potential participant to discuss the trial further and give them a participant information sheet.

If potential participants are interested in taking part in the study then they will be invited to attend a screening appointment with a member of the local research team (either in the clinic or home, by telephone, or by videoconference, depending on patient preference). Eligibility will be determined during the screening appointment. All eligible participants will be invited to provide fully informed written consent, verbal consent (for those who cannot provide written consent due to mobility difficulties or if verbal consent is being obtained by telephone or videoconference due to COVID-19 related restrictions), or consent via the use of a communication aid to participate in the trial, in line with Sheffield Clinical Trials Research Unit’s (CTRU) standard operating procedures (SOPs). An independent witness will be asked to sign the consent form to verify the consent taken in all cases where non-written consent is obtained in-person. In cases where verbal consent is obtained by telephone or videoconference, the conversation regarding consent will be audio recorded using an encrypted digital voice recorder, with the potential participant's agreement.

Therapists will be identified from the group of study therapists who will be involved in delivering the intervention to plwMND and approached by the trial’s research assistant. Other procedures will be similar to those described above.

### Randomisation

PlwMND will be allocated in equal proportions to one of two arms (ACT plus UC or UC alone) using a computer generated pseudo-random list. Randomisation will use blocks of varying length, stratified by recruitment site. The allocation sequence will be hosted by Sheffield CTRU in accordance with their SOPs and will be held on a secure server. Access to the concealed allocation sequence will be restricted to those with authorisation. A CTRU statistician will supply the allocation ratio (1:1) and block sizes to Sheffield CTRU’s bespoke online randomisation system (SCRAM). Eligible plwMND will be randomised once they have provided fully informed consent and baseline measures have been collected.

### Blinding

Trial statisticians will be blinded to allocation as per Sheffield CTRU SOPs. The outcome assessor will be intended to be blind to treatment allocation for the duration of the trial, while plwMND, carers, study therapists and clinicians will be aware of this. The Data Monitoring and Ethics Committee (DMEC) will have access to unblinded data at their request during the trial. Any instances of accidental unblinding will be recorded at 6 and 9 months, when the outcome assessors are asked to guess the allocated arm for each participant with MND.

### Data collection

Participants will be asked to provide fully informed consent prior to any data collection. Socio-demographic and clinical data will be collected at screening and baseline. Following confirmation of eligibility, the majority of outcome measures will be completed at baseline (0 months), 6 months post-randomisation (± 4 weeks), and 9 months post-randomisation (± 4 weeks). Exceptions to this are shown in Table 1. Data collection will be conducted via telephone, videoconference, post, email, online or via face-to-face interview by a blind outcome assessor. Mode of administration will be recorded as this may impact on the collection of some outcome measures. A range of strategies will be used to promote participant retention, including provision of flexible means to participate, newsletters and non-contingent vouchers for completion of follow-up outcome measures.

**Table 1:**
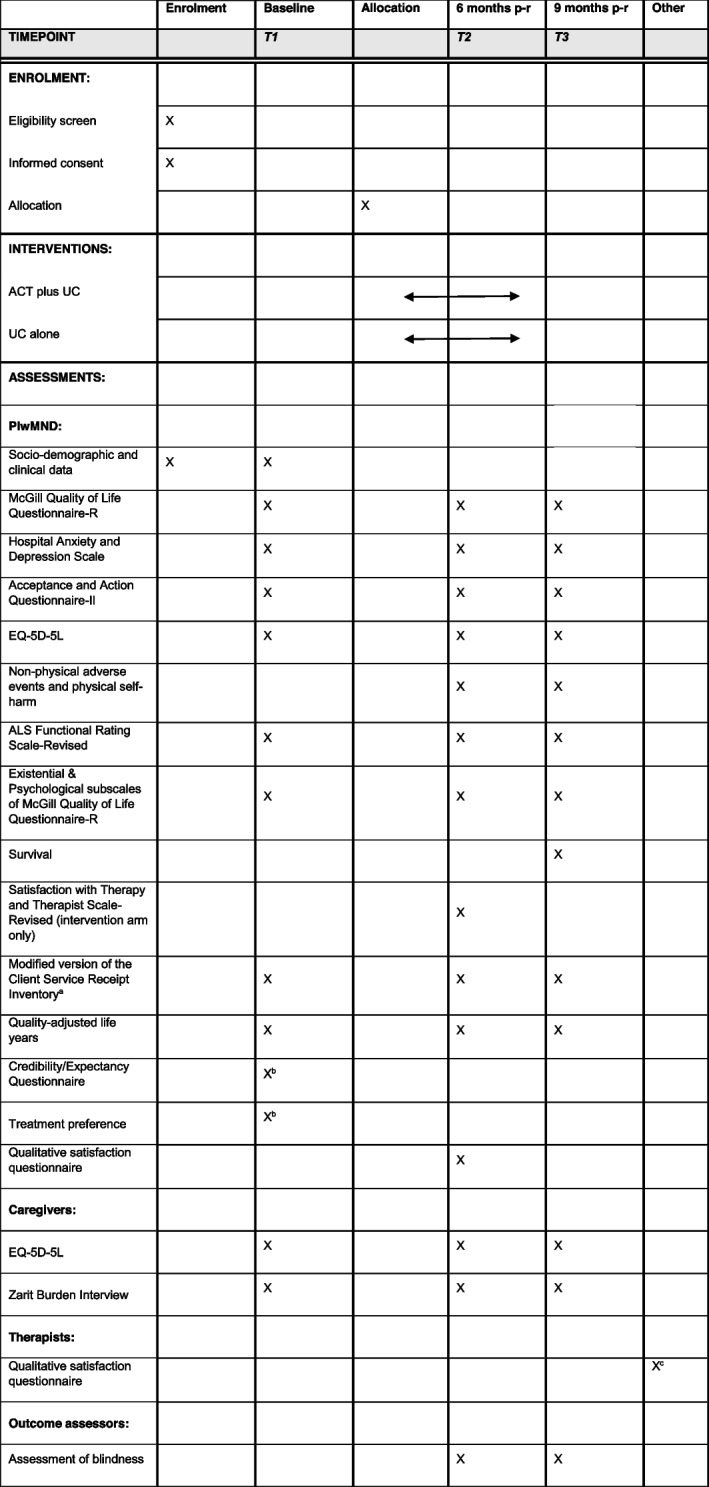
Schedule of enrolment, interventions and assessments

*ACT* Acceptance and Commitment Therapy, *ALS* amyotrophic lateral sclerosis, *p-r* post-randomisation, *UC* usual multidisciplinary care

a In order to avoid unblinding of outcome assessors, a question about the use of psychological therapies on the modified CSRI will be administered via post at 6 months and via the blinded outcome assessor at 9 months, after they have completed the unblinding question

b Completed after consent, but prior to randomisation, after participants are given a rationale for ACT

c Completed by the therapist at the end of their involvement in the trial

### Data management

Sheffield CTRU will oversee data collection, management and analysis and ensure the trial is undertaken according to Good Clinical Practice Guidelines and CTRU SOPs. Data will be collected and retained in accordance with The General Data Protection Regulation 2016/679. Participants will be assigned unique identification numbers and case report forms will not bear personal identifiable information apart from date of birth. Confidentiality will be maintained unless there is evidence of risk of harm to self or others. Data will be entered on a study database hosted on CTRU’s web-based data management system, Prospect. Prospect stores all data in a PostgreSQL database on virtual servers hosted by Corporate Information and Computing Services (CiCS) at the University of Sheffield. Prospect uses industry standard techniques to provide security, including password authentication and encryption using SSL/TLS. Encrypted audio files of verbal consents and therapy sessions will be recorded using encrypted digital voice recorders and uploaded to a secure server within University College London’s Data Safe Haven, which satisfies the highest level of security requirements of NHS trusts. All source documents will be securely retained for a period of 10 years following completion of the study.

### Quantitative analysis

A statistical analysis plan that includes the health economic analysis will be developed a priori and reviewed and approved by the Trial Steering Committee (TSC). The primary outcome measure will be analysed using a multi-level mixed effects model in which treatment group and baseline score will be included as fixed effect covariates and therapist will be included as a random effect to account for potential clustering. Analyses will be conducted separately at 6 months (the primary analysis time point) and 9 months. The difference between groups in mean quality of life will be quantified by the model coefficient, along with its 95% confidence interval. Primary analyses will be by intention to treat, but additional sensitivity analyses will be used to assess whether outcomes vary across sites/therapists, and by disease severity at baseline, psychotropic medication use, number of weeks taken to complete the therapy sessions and participants’ engagement in the intervention (as determined by the number of sessions completed within 4 months, and if applicable, whether the sessions were ongoing beyond 6 months post-randomisation).

Secondary outcome measures (for patients and caregivers) will be analysed in a similar fashion to the primary outcome measure. Adverse events will be summarised as the number and percentage of patients experiencing each event and the number of events by treatment arm. Patient deaths are expected to be relatively uncommon at 9 months (< 10%) and will be summarised descriptively as an adverse event. It is expected that some participants will have missing outcome data either due to death, loss to follow up or withdrawal from trial. The number of missing values will be summarised by treatment group, time point and reason. Multiple imputation using Rubin's rules [64] will be implemented for the primary and other key endpoints if the level of missing data exceeds 5% for reasons other than participant death.

Additional exploratory analysis will also be undertaken to assess the consistency of treatment effect across the following subgroups: i) severity of depression and/or anxiety at baseline, according to MND-specific clinical cut-offs on the Hospital Anxiety and Depression Scale [51]; ii) patient preference for treatment; iii) use of pharmacological therapy for mood disorder; and iv) disease severity as measured using the ALS Functional Rating Scale-Revised. We will also undertake exploratory analyses in those who score below clinical cut-offs for anxiety or depression at baseline on the Hospital Anxiety and Depression Scale to see whether ACT is beneficial in preventing progression to clinical levels of these symptoms at follow-up. In addition, we will conduct subgroup analyses to examine the potential impact of the COVID-19 pandemic on the reliability of trial results (with cut points for before and after the beginning of national lockdown on 23 March 2020 and before and after the final easing of lockdown restrictions on 27 January 2022).

The impact of non-adherence (i.e. non-uptake of ACT in the intervention group) and contamination (i.e. delivery of psychological therapy in the control group) will be assessed using complier-average causal effect analysis and a per-protocol analysis. Average Causal Response analysis will be used to assess any incremental impacts of the number of ACT sessions received [65].

### Qualitative analysis

Qualitative data from the Satisfaction questionnaire will be transcribed and anonymised to maintain confidentiality. Data will then be analysed iteratively using focussed thematic analysis [66]. Three members of the research team will independently code initial data before constructing an analytic framework around: i) facilitators/barriers to engagement, previous experiences of psychotherapy, and adaptations to ACT for plwMND; and ii) the acceptability, relevance, perceived value and feasibility of delivering ACT to plwMND. The analytical framework will be applied to the remaining transcripts, with themes and subthemes refined as necessary.

### Economic evaluation

A cost-utility analysis will present the incremental costs per quality-adjusted life years from an NHS and social care perspective of plwMND receiving ACT plus UC compared to those receiving UC alone. Costs will be estimated for each participant with MND and will include costs for delivering the intervention (training and staff time for delivering the intervention, cost of materials) and primary and secondary health care usage. Data on health care resource use will be collected using the modified CSRI and will collect information on hospital, nursing home and hospice services, out-patient visits and day care, primary and community care services, and equipment obtained. Unit costs will be derived from appropriate national sources and will include NHS reference costs and Personal Social Service Research Unit costs [67, 68]. The standard version of the EQ-5D-5L will be used to collect utility values, which will be used to estimate quality-adjusted life years. These will be calculated using the area under the curve method. Where data on the EQ-5D-5L or resource use are missing, multiple imputation techniques will be implemented. Differences between costs and quality-adjusted life years in the two groups will be described and the incremental cost effectiveness ratio will be calculated. A trial-based analysis will be supplemented by an analysis using a simple decision analytic model (a Markov model), which will be used to estimate the cost effectiveness of the intervention over the lifetime of plwMND. The model will use transition states related to the severity of MND (mild, moderate, severe, terminal and death) and will use a two-month cycle. It will be based on previous models published in the literature. This will be populated using the trial data plus information from the literature where required. This analysis will allow the estimation of lifetime cost-effectiveness and associated cost-effectiveness acceptability curves through the use of probabilistic sensitivity analysis. Caregiver costs will be included in a secondary analysis which will take a wider perspective to include patient and caregiver burden. Sensitivity analysis will explore assumptions made around transition probabilities, costs and long-term survival estimates. Bootstrapping will be used to capture uncertainty around cost-effectiveness estimates.

### Patient and Public Involvement (PPI)

PPI members are and have been involved at all stages of the study. The intervention was initially developed through a combination of interviews and workshops comprising people with MND, caregivers of people with MND and MND healthcare professionals. Five interested plwMND and caregivers were invited to be members of the study’s Patient/Caregiver Advisory Group. The intervention has been developed and refined in close collaboration with them, and they have also advised on research management, trial documents and dissemination of findings. PPI members attend Patient/Caregiver Advisory Group and Trial Management Group meetings, while independent plwMND and representatives from the MND Association attend TSC meetings.

### Data monitoring

The study will be conducted in line with the Helsinki Declaration. University College London is the nominated sponsor and research governance will be led by the UCL/UCLH Joint Research Office (uclh.randd@nhs.net). The study will be conducted in accordance with the protocol, Good Clinical Practice and Sheffield CTRU SOPs. All trial related documentation will be made available on request to the sponsor, Health Research Authority, Research Ethics Committee and other relevant authorities. An independent TSC (comprising academic clinicians, a statistician, a health economist and PPI representatives) and an independent DMEC (comprising academic clinicians and a statistician) will meet every 6–12 months to review progress and monitor the trial. All safety data will be reviewed by the DMEC. There will be no planned interim analyses.

### Safety

Trial sites will report Adverse Events (AEs) and Serious Adverse Events (SAEs) in accordance with Sheffield CTRU SOPs. These will be reported at any stage of trial participation, as well as at 6- and 9-months follow-up. An AE will be defined as any untoward medical occurrence in a trial participant with MND. All incidents of non-physical AEs will be collected and recorded, while physical AEs other than physical self-harm will not be as they are expected in this population. All SAEs will be reported to Sheffield CTRU and the sponsor within 24 h of discovery at site: those deemed both “unexpected” and “related” to the intervention or trial will be reported to the REC within 15 days of being reported to the study team.

### Ethics

The study has been approved by the London Dulwich Research Ethics Committee, Health Research Authority, and Health and Care Research Wales (19/LO/0272). Recruitment will only commence at a site when site-specific confirmation of capacity and capability has been obtained and the green light has been issued by the sponsor. Participants with MND, their caregivers and therapists will be consented in line with Sheffield CTRU SOPs and the Mental Capacity Act (2005). All participants will be asked to provide fully informed written consent, verbal consent (for those who cannot provide written consent), or consent via the use of a communication aid to participate in the trial. Participants will be made aware that their participation is voluntary and that they may withdraw from the intervention and/or trial, should they wish, at any time, without it affecting their rights. Participants may be withdrawn from the trial whenever continued participation is no longer in their best interests. Any amendments will be approved by the sponsor and communicated to all sites and the Health Research Authority. Compensation to those who suffer harm from trial participation will be available through UCL insurance.

### Dissemination

Dissemination to the academic and clinical community, plwMND and their families, and the broader public will occur through peer-reviewed, international, open-access academic journals, academic conferences, local clinical conferences and meetings, talks to local MND support groups and the MND Association, social media (e.g. University media releases, University website and Twitter feeds), and ACT training and seminars. In addition, the trial results will be reported in the ISRCTN database, and participants who have indicated they would like a copy of the results will be sent a summary of the findings. Standard author eligibility guidelines will be followed.

## Conclusion

Clear evidence-based guidance regarding the psychological management of plwMND within the UK is currently lacking. This RCT will assess the clinical and cost effectiveness of modified ACT plus UC in comparison to UC alone for improving psychological health in plwMND. The application of an ACT intervention that has been specifically adapted to the unique psychological, physical and communication needs of plwMND is novel. To our knowledge, not only will this RCT be the first to evaluate ACT in this population, it will also be the largest clinical trial of a psychological intervention for plwMND to date. Consequently, findings from this trial will provide much needed guidance to UK MND Care Centres/clinics and Clinical Commissioners.

Limitations of the trial include the use of a non-active control arm, potential unblinding of outcome assessors and the relatively short follow-up period. The use of a non-active control arm (i.e. UC) means that it will not be possible to determine whether any potentially beneficial treatment effects are due to non-specific therapeutic factors such as social support. Furthermore, blinded outcome assessors may be inadvertently unblinded given that plwMND and caregivers will not be blinded to arm allocation, which may bias participant-reported outcomes. Study procedures, as outlined above, are in place to minimise the risk of unblinding and potential biasing of results. Another limitation is the relatively short follow-up period, since outcome measures will be collected at baseline and 6- and 9-months post-randomisation. Although these follow-up periods were chosen for pragmatic reasons based on typical life expectancies, it means that it will not be possible to examine longer-term treatment effects.

An important issue that will need to be considered when interpreting the findings of this trial is the impact of the COVID-19 pandemic on plwMND. We recently highlighted the negative impact of COVID-19 and related restrictions on this clinically vulnerable group, including reduced access to UC, increased rates of anxiety and increased social isolation due to shielding [69]. Although our intervention was originally developed so that it could be delivered remotely (e.g. via videoconference), all trial procedures have been adapted so that they can be conducted remotely in response to the COVID-19 pandemic. These changes, as well as changes to the context of the trial, will need to be carefully considered when analysing and interpreting trial data. Subgroup analyses examining pre-, during- and post-lockdown restrictions will be essential in examining the potential impact of the COVID-19 pandemic on trial outcomes.

## Supplementary Information

**Additional file 1.** WHO Trial Registration Data Set.

**Additional file 2.** Template for intervention description and replication (TIDieR) checklist.

**Additional file 3.** Outline of the ACT intervention adapted for plwMND.

**Additional file 4.** Example consent form.

## Data Availability

Access to quantitative datasets generated and/or analysed during this study will be included in subsequent publications of results, where they will be sufficiently de-identified for data-sharing and conform to ethics and data governance requirements. Qualitative datasets will not be shared as it will not be possible to de-identify these data sufficiently and retain data integrity. The full trial protocol is available at https://fundingawards.nihr.ac.uk/award/16/81/01. A model consent form is provided in Supplementary File 4. Participant information sheets are available at: https://www.ucl.ac.uk/psychiatry/research/mental-health-older-people/projects/commend/get-involved.

## Abbreviations

ACT: Acceptance and Commitment Therapy
AE: Adverse event
ALS: Amyotrophic lateral sclerosis
CBT: Cognitive Behavioural Therapy
CSRI: Client Service Receipt Inventory
CTRU: Clinical Trials Research Unit
DMEC: Data Monitoring and Ethics Committee
MND: Motor neuron disease
NIV: Non-invasive ventilation
PEG: Percutaneous endoscopic gastronomy
plwMND: People living with MND
PPI: Patient and public involvement
RCT: Randomised controlled trial
SAE: Serious adverse event
SOP: Standard operating procedure
TSC: Trial Steering Committee
UC: Usual multidisciplinary care

## References

[1] Al-Chalabi A, Hardiman O (2013). The epidemiology of ALS: a conspiracy of genes, environment and time. Nat Rev Neurol.

[2] Chiò A, Logroscino G, Hardiman O, Swingler R, Mitchell D, Beghi E (2009). Prognostic factors in ALS: a critical review. Amyotroph Lateral Scler.

[3] Turner MR, Parton MJ, Shaw CE, Leigh PN, Al-Chalabi A (2003). Prolonged survival in motor neuron disease: a descriptive study of the King’s database 1990–2002. J Neurol Neurosurg Psychiatry.

[4] Miller RG, Mitchell JD, Moore DH (2012). Riluzole for amyotrophic lateral sclerosis (ALS)/motor neuron disease (MND). Cochrane Database Syst Rev.

[5] Averill AJ, Kasarskis EJ, Segerstrom SC (2007). Psychological health in patients with amyotrophic lateral sclerosis. Amyotroph Lateral Scler.

[6] Kurt A, Nijboer F, Matuz T, Kübler A (2007). Depression and anxiety in individuals with amyotrophic lateral sclerosis: epidemiology and management. CNS Drugs.

[7] Taylor L, Wicks P, Leigh PN, Goldstein LH (2010). Prevalence of depression in amyotrophic lateral sclerosis and other motor disorders. Eur J Neurol.

[8] Fang F, Valdimarsdóttir U, Fürst CJ, Hultman C, Fall K, Sparén P (2008). Suicide among patients with amyotrophic lateral sclerosis. Brain.

[9] McDonald ER, Wiedenfeld SA, Hillel A, Carpenter CL, Walter RA (1994). Survival in amyotrophic lateral sclerosis. The role of psychological factors. Arch Neurol.

[10] Johnston M, Earll L, Giles M, Mcclenahan R, Stevens D, Morrison V (1999). Mood as a predictor of disability and survival in patients newly diagnosed with ALS/MND. Br J Health Psychol.

[11] Pizzimenti A, Aragona M, Onesti E, Inghilleri M (2013). Depression, pain and quality of life in patients with amyotrophic lateral sclerosis: a cross-sectional study. Funct Neurol.

[12] van Groenestijn AC, Kruitwagen-van Reenen ET, Visser-Meily JMA, van den Berg LH, Schröder CD (2016). Associations between psychological factors and health-related quality of life and global quality of life in patients with ALS: a systematic review. Health Qual Life Outcomes.

[13] National Institute for Health and Care Excellence. Motor neurone disease: Assessment and management (NG42) [Internet]. 2016. Available from: www.nice.org.uk/Guidance/NG42. 26962594

[14] National Institute for Health Care Excellence. Common mental health problems: Identification and pathways to care (CG123). 2016.

[15] Weeks KR, Gould RL, Mcdermott C, Lynch J, Goldstein LH, Graham CD (2019). Needs and preferences for psychological interventions of people with motor neuron disease. Amyotroph Lateral Scler Front Degener.

[16] Hayes S, Strosahl K, Wilson K (2012). Acceptance and Commitment Therapy: The process and practice of mindful change.

[17] McCracken LM, Yu L, Vowles KE (2022). New generation psychological treatments in chronic pain. BMJ.

[18] Gloster AT, Walder N, Levin ME, Twohig MP, Karekla M (2020). The empirical status of acceptance and commitment therapy: a review of meta-analyses. J Context Behav Sci.

[19] Hayes SC, Strosahl K, Wilson KG, Bissett RT, Pistorello J, Toarmino D (2004). Measuring experiential avoidance: a preliminary test of a working model. Psychol Rec.

[20] Kangas M, McDonald S (2011). Is it time to act? The potential of acceptance and commitment therapy for psychological problems following acquired brain injury. Neuropsychol Rehabil.

[21] Graham CD, Simmons Z, Stuart SR, Rose MR (2015). The potential of psychological interventions to improve quality of life and mood in muscle disorders. Muscle Nerve.

[22] Serfaty M. The addition of ACT or a Talking Control to treatment as usual for the management of dysfunction in advanced cancer: a feasibility randomised controlled trial (CanACT).

[23] Hadlandsmyth K, White KS, Nesin AE, Greco LA (2013). Proposing an acceptance and commitment therapy intervention to promote improved diabetes management in adolescents: a treatment conceptualization. Int J Behav Consult Ther.

[24] McCracken LM, Vowles KE (2014). Acceptance and commitment therapy and mindfulness for chronic pain: model, process, and progress. Am Psychol.

[25] Pagnini F, Phillips D, Bosma CM, Reece A, Langer E, Langer E (2015). Mindfulness, physical impairment and psychological well-being in people with amyotrophic lateral sclerosis. Psychol Health.

[26] Graham C. Explaining and changing adverse illness perceptions in people with muscle disease. Kings College London; 2012.

[27] Graham CD, Rose MR, Hankins M, Chalder T, Weinman J (2013). Separating emotions from consequences in muscle disease: Comparing beneficial and unhelpful illness schemata to inform intervention development. J Psychosom Res.

[28] Graham CD, Gouick J, Ferreira N, Gillanders D (2016). The influence of psychological flexibility on life satisfaction and mood in muscle disorders. Rehabil Psychol.

[29] Graham CD, Rose MR (2017). What explains high life satisfaction in men living with Duchenne muscular dystrophy? A preliminary study to inform psychological intervention. Muscle Nerve.

[30] Low J, Davis S, Drake R, King M, Tookman A, Turner K (2012). The role of acceptance in rehabilitation in life-threatening illness. J Pain Symptom Manage.

[31] Pakenham KI, Fleming M (2011). Relations between acceptance of multiple sclerosis and positive and negative adjustments. Psychol Health.

[32] Veehof MM, Trompetter HR, Bohlmeijer ET, Schreurs KMG (2016). Acceptance- and mindfulness-based interventions for the treatment of chronic pain: a meta-analytic review. Cogn Behav Ther.

[33] Clarke S, Kingston J, James K, Bolderston H, Remington B (2014). Acceptance and commitment therapy group for treatment-resistant participants: a randomized controlled trial. J Context Behav Sci.

[34] Wetherell JL, Liu L, Patterson TL, Afari N, Ayers CR, Thorp SR (2011). Acceptance and commitment therapy for generalized anxiety disorder in older adults: a preliminary report. Behav Ther.

[35] Gould RL, Rawlinson C, Thompson B, Weeks K, Gossage-Worrall R, Cantrill H, et al. Acceptance and Commitment Therapy for people living with motor neuron disease: A feasibility study. submitted; 10.1186/s40814-023-01354-7 PMC10327371 37420261

[36] Hoffmann TC, Glasziou PP, Boutron I, Milne R, Perera R, Moher D, et al. Better reporting of interventions: template for intervention description and replication (TIDieR) checklist and guide. BMJ [Internet]. 2014 Mar 7 [cited 2020 Jul 14];348. Available from: https://www.bmj.com/content/348/bmj.g1687. 10.1136/bmj.g1687 24609605

[37] Chan AW, Tetzlaff JM, Altman DG, Laupacis A, Gøtzsche PC, Krleža-Jerić K (2013). SPIRIT 2013 statement: defining standard protocol items for clinical trials. Ann Intern Med.

[38] Al-Chalabi A, Hardiman O, Kiernan MC, Chiò A, Rix-Brooks B, van den Berg LH (2016). Amyotrophic lateral sclerosis: moving towards a new classification system. Lancet Neurol.

[39] Brooks BR, Miller RG, Swash M, Munsat TL, World Federation of Neurology Research Group on Motor Neuron Diseases. El Escorial revisited: revised criteria for the diagnosis of amyotrophic lateral sclerosis. Amyotroph Lateral Scler Mot Neuron Disord Off Publ World Fed Neurol Res Group Mot Neuron Dis. 2000 Dec;1(5):293–9. 10.1080/146608200300079536 11464847

[40] Strong MJ, Abrahams S, Goldstein LH, Woolley S, Mclaughlin P, Snowden J (2017). Amyotrophic lateral sclerosis - frontotemporal spectrum disorder (ALS-FTSD): Revised diagnostic criteria. Amyotroph Lateral Scler Front Degener.

[41] McKhann GM, Knopman DS, Chertkow H, Hyman BT, Jack CR, Kawas CH (2011). The diagnosis of dementia due to Alzheimer’s disease: Recommendations from the National Institute on Aging-Alzheimer’s Association workgroups on diagnostic guidelines for Alzheimer’s disease. Alzheimers Dement.

[42] Beecham J, Knapp M. Costing psychiatric interventions. In: Measuring Mental Health Needs. London: Gaskell; 1992.

[43] Plumb JC, Vilardaga R (2010). Assessing treatment integrity in acceptance and commitment therapy: Strategies and suggestions. Int J Behav Consult Ther.

[44] Nezu A, Nezu C (2008). Evidence-based outcome research: A practical guide to conducting randomized controlled trials for psychosocial interventions.

[45] Cohen SR, Sawatzky R, Russell LB, Shahidi J, Heyland DK, Gadermann AM (2017). Measuring the quality of life of people at the end of life: The McGill Quality of Life Questionnaire-Revised. Palliat Med.

[46] Gagnon P, Fillion L, Robitaille MA, Girard M, Tardif F, Cochrane JP (2015). A cognitive–existential intervention to improve existential and global quality of life in cancer patients: A pilot study. Palliat Support Care.

[47] Cohen SR, Mount BM (2000). Living with cancer: ?Good? days and ?bad? days?What produces them?. Cancer.

[48] Burns TM, Graham CD, Rose MR, Simmons Z (2012). Quality of life and measures of quality of life in patients with neuromuscular disorders. Muscle Nerve.

[49] Simmons Z, Bremer BA, Robbins RA, Walsh SM, Fischer S (2000). Quality of life in ALS depends on factors other than strength and physical function. Neurology.

[50] Zigmond AS, Snaith RP (1983). The hospital anxiety and depression scale. Acta Psychiatr Scand.

[51] Gibbons CJ, Mills RJ, Thornton EW, Ealing J, Mitchell JD, Shaw PJ (2011). Rasch analysis of the hospital anxiety and depression scale (hads) for use in motor neurone disease. Health Qual Life Outcomes.

[52] Bond FW, Hayes SC, Baer RA, Carpenter KM, Guenole N, Orcutt HK (2011). Preliminary Psychometric Properties of the Acceptance and Action Questionnaire–II: a revised measure of psychological inflexibility and experiential avoidance. Behav Ther.

[53] Herdman M, Gudex C, Lloyd A, Janssen MF, Kind P, Parkin D (2011). Development and preliminary testing of the new five-level version of EQ-5D (EQ-5D-5L). Qual Life Res.

[54] Cedarbaum JM, Stambler N, Malta E, Fuller C, Hilt D, Thurmond B, et al. The ALSFRS-R: a revised ALS functional rating scale that incorporates assessments of respiratory function. BDNF ALS Study Group (Phase III). J Neurol Sci. 1999 Oct;169(1–2):13–21. 10.1016/s0022-510x(99)00210-5 10540002

[55] Oei TPS, Green AL (2008). The Satisfaction With Therapy and Therapist Scale-Revised (STTS-R) for group psychotherapy: Psychometric properties and confirmatory factor analysis. Prof Psychol Res Pract.

[56] Zarit SH, Reever KE, Bach-Peterson J (1980). Relatives of the impaired elderly: correlates of feelings of burden. Gerontologist.

[57] Devilly GJ, Borkovec TD (2000). Psychometric properties of the credibility/expectancy questionnaire. J Behav Ther Exp Psychiatry.

[58] Twohig MP, Hayes SC, Plumb JC, Pruitt LD, Collins AB, Hazlett-Stevens H (2010). A randomized clinical trial of acceptance and commitment therapy versus progressive relaxation training for obsessive-compulsive disorder. J Consult Clin Psychol.

[59] Lenglet T, Lacomblez L, Abitbol JL, Ludolph A, Mora JS, Robberecht W (2014). A phase II−III trial of olesoxime in subjects with amyotrophic lateral sclerosis. Eur J Neurol.

[60] Kuyken W, Byford S, Byng R, Dalgleish T, Lewis G, Taylor R (2010). Study protocol for a randomized controlled trial comparing mindfulness-based cognitive therapy with maintenance anti-depressant treatment in the prevention of depressive relapse/recurrence: the PREVENT trial. Trials.

[61] A-Tjak JGL, Davis ML, Morina N, Powers MB, Smits JAJ, Emmelkamp PMG. A Meta-Analysis of the Efficacy of Acceptance and Commitment Therapy for Clinically Relevant Mental and Physical Health Problems. Psychother Psychosom. 2015;84(1):30–6. 10.1159/000365764 25547522

[62] Graham CD, Gouick J, Krahé C, Gillanders D (2016). A systematic review of the use of Acceptance and Commitment Therapy (ACT) in chronic disease and long-term conditions. Clin Psychol Rev.

[63] Norman GR, Sloan JA, Wyrwich KW (2003). Interpretation of Changes in Health-related Quality of Life. Med Care.

[64] Little R, Rubin D (2002). Statistical Analysis with Missing Data (2nd edition).

[65] Little RJ, Long Q, Lin X (2009). A Comparison of methods for estimating the causal effect of a treatment in randomized clinical trials subject to noncompliance. Biometrics.

[66] Braun V, Clarke V (2006). Using thematic analysis in psychology. Qual Res Psychol.

[67] Department of Health. NHS reference costs 2014/2015. 2015.

[68] Curtis L, Burns A (2015). Unit costs of health and social care 2015.

[69] Musson LS, Collins A, Opie-Martin S, Bredin A, Hobson EV, Barkhouse E (2022). Impact of the covid-19 pandemic on amyotrophic lateral sclerosis care in the UK. Amyotroph Lateral Scler Front Degener.

