# Supplementary material for: A randomised controlled trial of acceptance and commitment therapy plus usual care compared to usual care alone for improving psychological health in people with motor neuron disease (COMMEND): study protocol
Source: BMC Neurol. 2022 Nov 15;22:431. doi: 10.1186/s12883-022-02950-5 (PMC9664029; doi:10.1186/s12883-022-02950-5)
Supplement: Supplementary file 1 — Additional file 1. WHO Trial Registration Data Set. [file 12883_2022_2950_MOESM1_ESM.docx]

Supplementary File 2: WHO Trial Registration Data Set.

| **Data category** | **Information** |
| --- | --- |
| Primary registry and trial identifying number | ISRCTN Registry, ISRCTN12655391, <https://www.isrctn.com/ISRCTN12655391> |
| Date of registration in primary registry | 17 July 2017 |
| Secondary identifying numbers | IRAS 255069, REC 19/LO/0272, NIHR HTA 16/81/01 |
| Source(s) of monetary or material support | 1. National Institute for Health and Care Research Health Technology Assessment Programme (16/81/01)  2. Motor Neurone Disease Association (Gould/Jul17/936-794) |
| Primary sponsor | University College London |
| Secondary sponsor(s) | N/A |
| Contact for public queries | Rebecca Gould (r.gould@ucl.ac.uk) |
| Contact for scientific queries | Rebecca Gould (r.gould@ucl.ac.uk) |
| Public title | A trial of Acceptance and Commitment Therapy for people with motor neuron disease |
| Scientific title | A feasibility study and randomised controlled trial of acceptance and COMmitment therapy for people with Motor nEuroN Disease (COMMEND) |
| Countries of recruitment | UK |
| Health condition(s) or problem(s) studied | Motor neuron disease |
| Intervention(s) | Acceptance and Commitment Therapy plus usual multidisciplinary care vs. usual multidisciplinary care alone |
| Key inclusion and exclusion criteria | *People living with MND:*  Inclusion criteria:   1. Aged ≥18 years. 2. Diagnosis of definite, laboratory-supported probable, clinically probable, or possible familial or sporadic ALS, progressive muscular atrophy or primary lateral sclerosis.   Exclusion criteria:   1. Current clinical need for any form of gastrostomy feeding or non-invasive ventilation. 2. Diagnosis of dementia using standard diagnostic guidelines. 3. Currently receiving ongoing formal psychological therapy delivered by a formally trained psychologist or psychotherapist, and unwilling to refrain from engaging in such formal psychological therapy during the receipt of ACT. 4. Insufficient understanding of English. 5. Lacking capacity to provide fully informed consent to participate in the trial. 6. A need for treatment for severe psychiatric disorder such as schizophrenia or bipolar disorder, or expressing suicidal ideation with active plans/suicidal behaviours and imminent intent. 7. Other medical factors that could compromise full study participation. 8. Previous participation in Phase 1 (an uncontrolled feasibility study).   *Caregivers of people living with MND:*  Inclusion criteria:   1. Aged ≥18 years. 2. Primary informal caregiver of a person with MND who has consented to participate in the trial.   *Therapists:*  Inclusion criteria:   1. Aged ≥18 years. 2. Study therapists who are involved in delivering the intervention in the trial. |
| Study type | Multi-centre, assessor-blind, parallel, two-arm randomised controlled trial |
| Date of first enrolment | 5 November 2019 |
| Target sample size | 188 |
| Recruitment status | Recruiting |
| Primary outcome(s) | McGill Quality of Life Questionnaire-R |
| Key secondary outcomes | *People living with MND:*   - Hospital Anxiety and Depression Scale - Acceptance and Action Questionnaire-II - EQ-5D-5L - Non-physical adverse events and physical self-harm - ALS Functional Rating Scale-Revised - Existential and Psychological subscales of the McGill Quality of Life Questionnaire-R - Survival at 9 months - Satisfaction with Therapy and Therapist Scale-Revised - Client Service Receipt Inventory - Quality-adjusted life years and resource use   *Caregivers of people living with MND:*   - EQ-5D-5L - Zarit Burden Interview |
