# Supplementary material for: A randomised controlled trial of acceptance and commitment therapy plus usual care compared to usual care alone for improving psychological health in people with motor neuron disease (COMMEND): study protocol
Source: BMC Neurol. 2022 Nov 15;22:431. doi: 10.1186/s12883-022-02950-5 (PMC9664029; doi:10.1186/s12883-022-02950-5)
Supplement: Supplementary file 2 — Additional file 2. Template for intervention description and replication (TIDieR) checklist. [file 12883_2022_2950_MOESM2_ESM.docx]

Supplementary File 3: Template for intervention description and replication (TIDieR) checklist.

| **Item** | **Section: page no./ paragraph no.** |
| --- | --- |
| **BRIEF NAME** |  |
| 1. Provide the name or a phrase that describes the intervention. | Introduction: p4/para 3 to p5/para 1, Methods: p8/para 3 |
| **WHY** |  |
| 2. Describe any rationale, theory, or goal of the elements essential to the intervention. | Introduction: p4/para 3 to p5/para 2 |
| **WHAT** |  |
| 3. Materials: Describe any physical or informational materials used in the intervention, including those provided to participants or used in intervention delivery or in training of intervention providers. Provide information on where the materials can be accessed (e.g. online appendix, URL). | Methods: p8/para 3 to p9/para 1 |
| 4. Procedures: Describe each of the procedures, activities, and/or processes used in the intervention, including any enabling or support activities. | Supplementary File 4 |
| **WHO PROVIDED** |  |
| 5. For each category of intervention provider (e.g. psychologist, nursing assistant), describe their expertise, background and any specific training given. | Methods: p8/para 4 to p9/para 1 |
| **HOW** |  |
| 6. Describe the modes of delivery (e.g. face-to-face or by some other mechanism, such as internet or telephone) of the intervention and whether it was provided individually or in a group. | Methods: p8/para 3, Supplementary File 4 |
| **WHERE** |  |
| 7. Describe the type(s) of location(s) where the intervention occurred, including any necessary infrastructure or relevant features. | Methods: p8/para 3 |
| **WHEN and HOW MUCH** |  |
| 8. Describe the number of times the intervention was delivered and over what period of time including the number of sessions, their schedule, and their duration, intensity or dose. | Methods: p8/para 3, Supplementary File 4 |
| **TAILORING** |  |
| 9. If the intervention was planned to be personalised, titrated or adapted, then describe what, why, when, and how. | Supplementary File 4 |
| **MODIFICATIONS** |  |
| 10. If the intervention was modified during the course of the study, describe the changes (what, why, when, and how). | N/A |
| **HOW WELL** |  |
| 11. Planned: If intervention adherence or fidelity was assessed, describe how and by whom, and if any strategies were used to maintain or improve fidelity, describe them. | Methods: p10/para 2 |
| 12. Actual: If intervention adherence or fidelity was assessed, describe the extent to which the intervention was delivered as planned. | N/A |
