# Supplementary material for: A randomised controlled trial of acceptance and commitment therapy plus usual care compared to usual care alone for improving psychological health in people with motor neuron disease (COMMEND): study protocol
Source: BMC Neurol. 2022 Nov 15;22:431. doi: 10.1186/s12883-022-02950-5 (PMC9664029; doi:10.1186/s12883-022-02950-5)
Supplement: Supplementary file 3 — Additional file 3. Outline of the ACT intervention adapted for plwMND. [file 12883_2022_2950_MOESM3_ESM.docx]

Supplementary File 4: Outline of the ACT intervention adapted for plwMND^a^.

| **Session** | **Main focus of the session** |
| --- | --- |
| 1 | Assessment of current issues and aims of therapy and introduction to ACT. |
| 2-7^b^ | 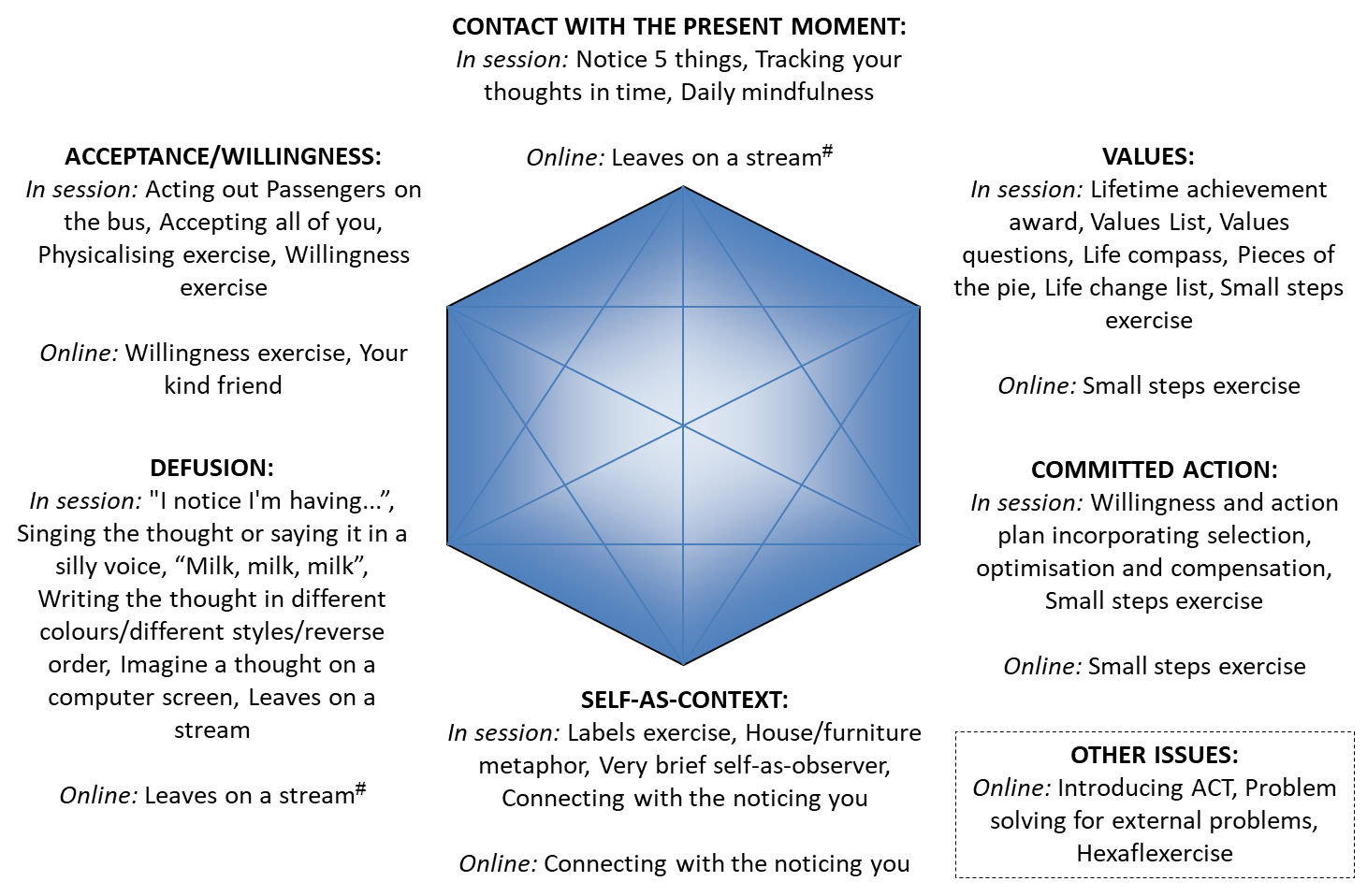 |
| 8 | Review skills and concepts, and ways of handling symptoms in the future. |

*Notes:*

^a^ The intervention has been developed so that it can be tailored to the psychological, physical, communication and cognitive needs of people with MND. Modules focus on the six core evidence-based processes of psychological flexibility as a basis for improving daily performance and wellbeing. Each module has been associated with a set of skills, metaphors, experiential exercises and homework tasks specifically adapted for people with MND and designed to increase psychological flexibility. The intervention also incorporates an initial assessment aimed at developing a shared understanding of a person's current difficulties within an ACT framework, and relapse prevention aimed at reviewing any gains made and ways of maintaining these. In addition, the intervention has been adjusted to accommodate physical and communication difficulties by drawing on theoretical principles of 'Selective Optimisation with Compensation. These involve strategies for helping people to choose the best functional domains to focus their resources on, engage in tasks that they perform best, and find ways of compensating for losses. The intervention also addresses mild cognitive difficulties (predominantly involving executive or language dysfunction) as these have been reported in approximately 50% of people with MND. Standard therapeutic strategies have been used to compensate for communication issues and mild cognitive difficulties such as working with communication aids, providing a workbook and session summaries as a reminder of the content of the sessions, clarifying and repeating key concepts and skills within and between sessions (e.g. recapping on the previous session at the beginning of the next session), working at a slower pace, and providing appointment reminders. Finally, it has been ensured that the intervention is relevant to all participants and not just those experiencing symptoms of depression and/or anxiety by maintaining a focus on helping people with MND to participate as fully as possible in their lives in meaningful ways, in keeping with the overall aim of ACT, rather than on reducing symptoms of depression and/or anxiety.

^b^ Therapists have flexibility in deciding the order of delivery of sessions 2-7, based on client need and the case conceptualisation. In addition, they have a choice about which and how many metaphors and experiential exercises should be delivered in each session. This means that the pace of the sessions can be modified depending on the needs and abilities of the individual client. In addition, therapists are encouraged to help clients customise metaphors, experiential exercises and questions to their own unique experiences, as well as adapting any suggested phrasing to terminology they are comfortable and familiar with. This balances allowing therapists to have sufficient psychological flexibility to respond to clients' idiosyncratic presentations with ensuring that therapists have sufficient confidence, expertise and experience in delivering ACT.
