# Supplementary material for: A randomised controlled trial of acceptance and commitment therapy plus usual care compared to usual care alone for improving psychological health in people with motor neuron disease (COMMEND): study protocol
Source: BMC Neurol. 2022 Nov 15;22:431. doi: 10.1186/s12883-022-02950-5 (PMC9664029; doi:10.1186/s12883-022-02950-5)
Supplement: Supplementary file 4 — Additional file 4. Example consent form. [file 12883_2022_2950_MOESM4_ESM.doc]

#
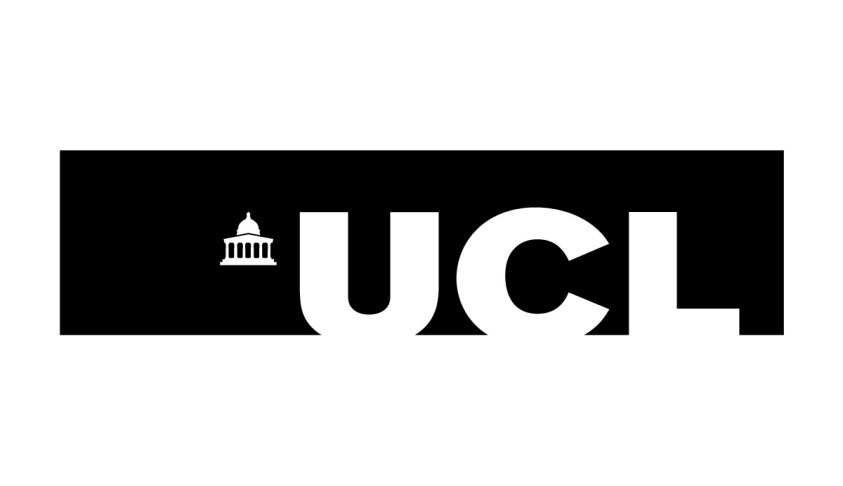


Site logo to be added

# **Consent Form for Patients** Participant Identification Number: ____/_____

A Randomised Controlled Trial of Acceptance and Commitment Therapy for people with motor neuron disease (COMMEND)

🞏 Please tick this box if consent was audio recorded

Please initial

the boxes

| 1. I have read the Information Sheet [insert version and date] for the above trial. I have been given a copy of the document to keep. |  |
| --- | --- |
| 1. I have had the opportunity to consider the information and ask questions about the above trial. I am satisfied with the responses I received to any questions I asked. |  |
| 1. I understand that my GP/MND Care Team will be informed of my participation in the trial. I agree to any necessary exchange of information between my GP and the research team. 2. I understand that my GP/MND Care Team will be contacted should any concerns arise during the course of the trial. 3. I understand that my MND Care Team will be contacted (if necessary) and/or relevant authorities will be notified (if necessary) if I disclose any actual or potentially illegal behaviour at any point during the trial. |  |
| 1. I understand that my participation is voluntary and that I am free to withdraw from the trial at any time, without having to give a reason, and without my medical care or legal rights being affected. 2. I understand that I will be randomised using a web-based system to either Acceptance and Commitment Therapy plus usual care (intervention group) or usual care alone (control group). 3. I understand that all of my therapy sessions will be audio recorded, if allocated to the intervention group. The audio recording of my sessions can be stopped at any time should I choose not to have a particular part of the discussion recorded. 4. I understand that if I verbally complete the satisfaction questionnaire at the end of the study with a member of the research team, this will be audio recorded. The audio recording will be transcribed and used in the research process. My name will not be included on the transcripts. 5. I understand that relevant sections of my medical notes and data collected during the trial may be looked at by individuals from University College London, from regulatory authorities, from the NHS Trust and the University of Sheffield, where it is relevant to my taking part in this research. I give permission for these individuals to have access to my records. 6. I understand that my data will be retained if I lose capacity to consent during the trial. 7. I understand that my caregiver (if applicable) does not have to take part in the trial if they do not want to, and their decision will not affect my taking part. I consent for my caregiver to be approached about taking part in the trial. 8. I give permission for my name, telephone number, postal address and email to be stored on the University of Sheffield database called Prospect.      1. I agree that information collected by the research team, including a copy of this signed consent form, can be sent securely to and stored at the Sheffield Clinical Trials Research Unit for the purposes of monitoring and auditing. 2. I agree to take part in the above trial.   **Optional statement (you do not have to initial this box if you do not agree with this statement – this will not affect whether you are eligible to take part in the above study):**   1. I understand that data collected about me, not containing personal identifiers may be used to support other ethically approved research studies in the future, and may be shared with other researchers for ethically approved comparison studies. |  |

🞏 Please tick this box if you would like a copy of the trial report.

Name of Participant (IN CAPITALS) ………………….….………………………………………………...

Participant’s signature .............................................… Date: .....................................

*Name of Independent Witness (IN CAPITALS) ………………….….………………………………………………...

Independent Witness’ signature .............................................… Date: .....................................

*An independent witness is only required to sign the consent form to verify consent provided a) in cases where non-written consent is obtained face-to-face b) specific instances where consent is obtained by telephone or videoconference.

🞏 Please tick this box to confirm that the independent witness is **NOT** the participating caregiver

🞏 Please tick this box to confirm that the independent witness is **NOT** a member of the COMMEND research team.

The Researcher has explained the trial to the Participant and has answered the Participant’s questions honestly and fully.

Name of Researcher (IN CAPITALS) ……………………………….……………………………………....

Researcher’s signature ...........................................… Date: ......................................

| This trial is funded by the NIHR Health Technology Assessment (HTA) Programme (ref: 16/81/01). The views expressed are those of the author(s) and not necessarily those of the NHS, the NIHR or the Department of Health. | 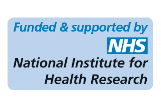 |
| --- | --- |
